# Supplementary material for: Repeated introductions and intensive community transmission fueled a mumps virus outbreak in Washington State
Source: eLife. 2021 Apr 19;10:e66448. doi: 10.7554/eLife.66448 (PMC8079146; doi:10.7554/eLife.66448)
Supplement: Supplementary file 1. — All genomes generated for this analysis are described above. Dates are formatted as year-month-day. Vaccination status, Ct, and sample collection type are all available for the Washington samples. Genome coverage represents the total proportion of bases in the genome with at least 20× coverage for which we were able to call a base. Sites with <20× coverage were labeled as Ns. Only samples with at least 50% non-N bases were included in the analysis. [file elife-66448-supp1.docx]

**Supplementary File 1a: Sample metadata**

| Strain name | Collection date | Sample type | Country | US state | Mumps genotype | Vaccination status | Cycle threshold (Ct) | Genome coverage |
| --- | --- | --- | --- | --- | --- | --- | --- | --- |
| MuVs/Washington.USA/48.16/FH1[G] | 2016-12-07 | Buccal Swab | USA | Washington | G | up-to-date | 29.32 | 0.91 |
| MuVs/Washington.USA/50.16/FH2[G] | 2016-12-13 | Buccal Swab | USA | Washington | G | up-to-date | 25.11 | 0.93 |
| MuVs/Washington.USA/50.16/FH3[G] | 2016-12-12 | Buccal Swab | USA | Washington | G | up-to-date | 28.53 | 0.84 |
| MuVs/Washington.USA/50.16/FH4[G] | 2016-12-13 | Buccal Swab | USA | Washington | G | unknown | 27.51 | 0.84 |
| MuVs/Washington.USA/1.17/FH5[G] | 2017-01-03 | Buccal Swab | USA | Washington | G | up-to-date | 23.04 | 0.93 |
| MuVs/Washington.USA/1.17/FH6[G] | 2017-01-04 | Buccal Swab | USA | Washington | G | not up-to-date | 32.87 | 0.93 |
| MuVs/Washington.USA/1.17/FH7[G] | 2017-01-06 | Buccal Swab | USA | Washington | G | up-to-date | 25.29 | 0.92 |
| MuVs/Washington.USA/1.17/FH8[G] | 2017-01-05 | Buccal Swab | USA | Washington | G | unknown | 26.61 | 0.93 |
| MuVs/Washington.USA/5.17/FH9[G] | 2017-01-31 | Buccal Swab | USA | Washington | G | up-to-date | 30.38 | 0.84 |
| MuVs/Washington.USA/5.17/FH10[G] | 2017-02-04 | Buccal Swab | USA | Washington | G | up-to-date | 26.72 | 0.92 |
| MuVs/Washington.USA/5.17/FH11[G] | 2017-02-08 | Buccal Swab | USA | Washington | G | up-to-date | 28.17 | 0.93 |
| MuVs/Washington.USA/6.17/FH12[G] | 2017-02-08 | Buccal Swab | USA | Washington | G | unknown | 25.00 | 0.93 |
| MuVs/Washington.USA/6.17/FH13[G] | 2017-02-13 | Buccal Swab | USA | Washington | G | up-to-date | 25.31 | 0.93 |
| MuVs/Washington.USA/6.17/FH14[G] | 2017-02-08 | Buccal Swab | USA | Washington | G | unknown | 29.04 | 0.93 |
| MuVs/Washington.USA/7.17/FH15[G] | 2017-02-15 | Buccal Swab | USA | Washington | G | unknown | 33.33 | 0.92 |
| MuVs/Washington.USA/9.17/FH16[G] | 2017-02-28 | Buccal Swab | USA | Washington | G | up-to-date | 23.17 | 0.93 |
| MuVs/Washington.USA/8.17/FH17[G] | 2017-02-23 | Buccal Swab | USA | Washington | G | up-to-date | 28.83 | 0.92 |
| MuVs/Washington.USA/9.17/FH18[G] | 2017-03-01 | Buccal Swab | USA | Washington | G | up-to-date | 31.84 | 0.84 |
| MuVs/Washington.USA/8.17/FH19[G] | 2017-02-28 | Buccal Swab | USA | Washington | G | unknown | 32.16 | 0.93 |
| MuVs/Washington.USA/15.17/FH20[G] | 2017-04-13 | Buccal Swab | USA | Washington | G | up-to-date | 22.25 | 0.93 |
| MuVs/Washington.USA/2.17/FH21[G] | 2017-01-13 | Buccal Swab | USA | Washington | G | up-to-date | 26.50 | 0.92 |
| MuVs/Washington.USA/2.17/FH22[G] | 2017-01-17 | Buccal Swab | USA | Washington | G | not up-to-date | 31.09 | 0.85 |
| MuVs/Washington.USA/2.17/FH23[G] | 2017-01-13 | Buccal Swab | USA | Washington | G | up-to-date | 25.61 | 0.92 |
| MuVs/Washington.USA/3.17/FH24[G] | 2017-01-19 | Buccal Swab | USA | Washington | G | up-to-date | 21.89 | 0.93 |
| MuVs/Washington.USA/3.17/FH25[G] | 2017-01-20 | Buccal Swab | USA | Washington | G | up-to-date | 27.23 | 0.93 |
| MuVs/Washington.USA/2.17/FH26[G] | 2017-01-12 | Buccal Swab | USA | Washington | G | up-to-date | 29.08 | 0.93 |
| MuVs/Washington.USA/5.17/FH27[G] | 2017-01-30 | Buccal Swab | USA | Washington | G | up-to-date | 24.53 | 0.93 |
| MuVs/Washington.USA/52.16/FH28[G] | 2016-12-31 | Buccal Swab | USA | Washington | G | unknown | 34.28 | 0.74 |
| MuVs/Washington.USA/1.17/FH29[G] | 2017-01-10 | Buccal Swab | USA | Washington | G | up-to-date | 33.14 | 0.64 |
| MuVs/Washington.USA/5.17/FH30[G] | 2017-02-02 | Buccal Swab | USA | Washington | G | unknown | 33.27 | 0.63 |
| MuVs/Washington.USA/9.17/FH31[G] | 2017-03-01 | Buccal Swab | USA | Washington | G | up-to-date | 34.94 | 0.66 |
| MuVs/Washington.USA/30.17/FH32[G] | 2017-07-25 | Buccal Swab | USA | Washington | G | not up-to-date | 21.88 | 0.93 |
| MuVs/Washington.USA/50.16/FH33[G] | 2016-12-19 | Buccal Swab | USA | Washington | G | unknown | 23.94 | 0.92 |
| MuVs/Washington.USA/12.17/FH34[G] | 2017-03-22 | Buccal Swab | USA | Washington | G | up-to-date | 24.15 | 0.93 |
| MuVs/Washington.USA/12.17/FH35[G] | 2017-03-24 | Buccal Swab | USA | Washington | G | not up-to-date | 28.12 | 0.93 |
| MuVs/Washington.USA/16.17/FH36[G] | 2017-04-19 | Buccal Swab | USA | Washington | G | up-to-date | 25.15 | 0.93 |
| MuVs/Washington.USA/17.17/FH37[G] | 2017-05-02 | Buccal Swab | USA | Washington | G | unknown | 25.71 | 0.93 |
| MuVs/Washington.USA/17.17/FH38[G] | 2017-04-25 | Buccal Swab | USA | Washington | G | unknown | 26.45 | 0.93 |
| MuVs/Washington.USA/17.17/FH39[G] | 2017-04-27 | Buccal Swab | USA | Washington | G | up-to-date | 26.69 | 0.93 |
| MuVs/Washington.USA/17.17/FH40[G] | 2017-04-29 | Buccal Swab | USA | Washington | G | up-to-date | 21.78 | 0.93 |
| MuVs/Washington.USA/51.16/FH41[G] | 2016-12-21 | Buccal Swab | USA | Washington | G | unknown | 23.56 | 0.93 |
| MuVs/Washington.USA/9.17/FH42[G] | 2017-03-06 | Buccal Swab | USA | Washington | G | up-to-date | 23.29 | 0.93 |
| MuVs/Washington.USA/10.17/FH43[G] | 2017-03-07 | Buccal Swab | USA | Washington | G | up-to-date | 24.50 | 1.00 |
| MuVs/Washington.USA/19.17/FH44[G] | 2017-05-10 | Buccal Swab | USA | Washington | G | unknown | 27.26 | 0.93 |
| MuVs/Washington.USA/49.16/FH45[G] | 2016-12-10 | Buccal Swab | USA | Washington | G | unknown | 30.24 | 0.93 |
| MuVs/Washington.USA/51.16/FH46[G] | 2016-12-22 | Buccal Swab | USA | Washington | G | unknown | 25.69 | 0.93 |
| MuVs/Washington.USA/2.17/FH47[G] | 2017-01-11 | Buccal Swab | USA | Washington | G | up-to-date | 24.04 | 0.93 |
| MuVs/Washington.USA/4.17/FH48[G] | 2017-01-28 | Buccal Swab | USA | Washington | G | up-to-date | 20.15 | 0.93 |
| MuVs/Washington.USA/4.17/FH49[G] | 2017-01-31 | Buccal Swab | USA | Washington | G | up-to-date | 31.31 | 0.93 |
| MuVs/Washington.USA/5.17/FH50[G] | 2017-02-06 | Buccal Swab | USA | Washington | G | unknown | 31.14 | 0.85 |
| MuVs/Washington.USA/6.17/FH51[G] | 2017-02-06 | Buccal Swab | USA | Washington | G | unknown | 26.59 | 0.92 |
| MuVs/Washington.USA/10.17/FH52[G] | 2017-03-12 | Buccal Swab | USA | Washington | G | not up-to-date | 23.76 | 0.93 |
| MuVs/Washington.USA/19.17/FH53[G] | 2017-05-12 | Buccal Swab | USA | Washington | G | unknown | 22.25 | 0.93 |
| MuVs/Washington.USA/2.17/FH54[G] | 2017-01-12 | Buccal Swab | USA | Washington | G | up-to-date | 24.47 | 0.84 |
| MuVs/Washington.USA/49.16/FH55[G] | 2016-12-07 | nasopharyngeal/throat swab | USA | Washington | G | unknown | 28.39 | 0.84 |
| MuVs/Washington.USA/11.17/FH56[G] | 2017-03-15 | Buccal Swab | USA | Washington | G | unknown | 25.31 | 1.00 |
| MuVs/Washington.USA/12.17/FH57[G] | 2017-03-22 | Buccal Swab | USA | Washington | G | unknown | 32.79 | 0.93 |
| MuVs/Washington.USA/2.17/FH58[G] | 2017-01-13 | Buccal Swab | USA | Washington | G | up-to-date | 23.20 | 0.93 |
| MuVs/Washington.USA/3.17/FH59[G] | 2017-01-18 | Buccal Swab | USA | Washington | G | up-to-date | 26.24 | 0.93 |
| MuVs/Washington.USA/4.17/FH60[G] | 2017-02-03 | Buccal Swab | USA | Washington | G | not up-to-date | 26.87 | 0.93 |
| MuVs/Washington.USA/7.17/FH61[G] | 2017-02-22 | Buccal Swab | USA | Washington | G | unknown | 24.86 | 0.98 |
| MuVs/Washington.USA/3.17/FH62[G] | 2017-01-19 | Buccal Swab | USA | Washington | G | unknown | 26.99 | 0.94 |
| MuVs/Washington.USA/16.17/FH63[G] | 2017-04-21 | Buccal Swab | USA | Washington | G | up-to-date | 24.26 | 0.99 |
| MuVs/Washington.USA/50.16/FH64[G] | 2016-12-12 | Buccal Swab | USA | Washington | G | up-to-date | 34.54 | 0.93 |
| MuVs/Washington.USA/1.17/FH65[G] | 2017-01-08 | Buccal Swab | USA | Washington | G | up-to-date | 28.53 | 0.93 |
| MuVs/Washington.USA/2.17/FH66[G] | 2017-01-13 | Buccal Swab | USA | Washington | G | unknown | 23.51 | 0.93 |
| MuVs/Washington.USA/3.17/FH67[G] | 2017-01-17 | Buccal Swab | USA | Washington | G | up-to-date | 29.70 | 0.93 |
| MuVs/Washington.USA/4.17/FH68[G] | 2017-01-26 | Buccal Swab | USA | Washington | G | up-to-date | 27.07 | 0.93 |
| MuVs/Washington.USA/21.17/FH69[G] | 2017-05-22 | Buccal Swab | USA | Washington | G | not up-to-date | 22.46 | 0.93 |
| MuVs/Washington.USA/4.17/FH70[G] | 2017-01-30 | Buccal Swab | USA | Washington | G | unknown | 25.32 | 0.93 |
| MuVs/Washington.USA/4.17/FH71[G] | 2017-01-30 | Buccal Swab | USA | Washington | G | up-to-date | 25.92 | 0.93 |
| MuVs/Washington.USA/5.17/FH72[G] | 2017-02-01 | Buccal Swab | USA | Washington | G | unknown | 22.74 | 0.93 |
| MuVs/Washington.USA/20.17/FH73[G] | 2017-05-21 | Buccal Swab | USA | Washington | G | up-to-date | 28.59 | 0.93 |
| MuVs/Washington.USA/22.17/FH74[G] | 2017-05-30 | Buccal Swab | USA | Washington | G | up-to-date | 28.84 | 0.93 |
| MuVs/Washington.USA/52.16/FH75[G] | 2016-12-30 | Buccal Swab | USA | Washington | G | up-to-date | 27.02 | 0.93 |
| MuVs/Washington.USA/12.17/FH76[G] | 2017-03-22 | Buccal Swab | USA | Washington | G | not up-to-date | 25.15 | 0.93 |
| MuVs/Washington.USA/1.17/FH77[G] | 2017-01-11 | Buccal Swab | USA | Washington | G | up-to-date | 34.73 | 0.92 |
| MuVs/Washington.USA/12.17/FH78[G] | 2017-03-24 | Buccal Swab | USA | Washington | G | unknown | 27.05 | 0.90 |
| MuVs/Washington.USA/16.17/FH79[G] | 2017-04-23 | Buccal Swab | USA | Washington | G | up-to-date | 27.44 | 0.93 |
| MuVs/Washington.USA/19.17/FH80[G] | 2017-05-11 | Buccal Swab | USA | Washington | G | unknown | 29.00 | 0.93 |
| MuVs/Washington.USA/20.17/FH81[G] | 2017-05-21 | Buccal Swab | USA | Washington | G | not up-to-date | 27.85 | 0.93 |
| MuVs/Washington.USA/20.17/FH82[G] | 2017-05-22 | Buccal Swab | USA | Washington | G | up-to-date | 33.04 | 0.84 |
| MuVs/Washington.USA/29.17/FH83[G] | 2017-07-26 | Buccal Swab | USA | Washington | G | not up-to-date | 32.28 | 0.93 |
| MuVs/Washington.USA/2.17/FH84[G] | 2017-01-13 | Buccal Swab | USA | Washington | G | not up-to-date | 23.08 | 0.84 |
| MuVs/Wisconsin.USA/16.14/FH85[G] | 2014-04 | unknown | USA | Wisconsin | G | unknown | unknown | 0.76 |
| MuVs/Wisconsin.USA/20.14/FH86[G] | 2014-05 | unknown | USA | Wisconsin | G | unknown | unknown | 0.58 |
| MuVs/Wisconsin.USA/15.06/FH87[H] | 2006-04 | unknown | USA | Wisconsin | H | unknown | unknown | 0.53 |
| MuVs/Missouri.USA/32.16/FH88[G] | 2016-08 | unknown | USA | Missouri | G | unknown | unknown | 0.78 |
| MuVs/Wisconsin.USA/15.17/FH89[G] | 2017-04 | unknown | USA | Wisconsin | G | unknown | unknown | 0.78 |
| MuVs/Alabama.USA/11.17/FH90[G] | 2017-03 | unknown | USA | Alabama | G | unknown | unknown | 0.80 |
| MuVs/Wisconsin.USA/15.06/FH91[A] | 2006-04 | unknown | USA | Wisconsin | A | unknown | unknown | 0.71 |
| MuVs/Alabama.USA/19.17/FH92[G] | 2017-05 | unknown | USA | Alabama | G | unknown | unknown | 0.77 |
| MuVs/Wisconsin.USA/37.06/FH93[G] | 2006-09 | unknown | USA | Wisconsin | G | unknown | unknown | 0.69 |
| MuVs/Washington.USA/9.17/FH94[K] | 2017-02-28 | Buccal Swab | USA | Washington | K | unknown | 29.13 | 0.63 |
| MuVs/Washington.USA/11.17/FH95[G] | 2017-03-15 | Buccal Swab | USA | Washington | G | up-to-date | 28.52 | 0.77 |
| MuVs/Washington.USA/11.17/FH96[G] | 2017-03-18 | Buccal Swab | USA | Washington | G | up-to-date | 36.91 | 0.76 |
| MuVs/Washington.USA/12.17/FH97[G] | 2017-03-23 | Buccal Swab | USA | Washington | G | up-to-date | 35.13 | 0.63 |
| MuVs/Washington.USA/14.17/FH98[G] | 2017-04-06 | Buccal Swab | USA | Washington | G | not up-to-date | 29.59 | 0.76 |
| MuVs/Wisconsin.USA/41.06/FH99[G] | 2006-10 | unknown | USA | Wisconsin | G | unknown | unknown | 0.77 |
| MuVs/Washington.USA/19.17/FH100[G] | 2017-05-11 | Buccal Swab | USA | Washington | G | up-to-date | 29.44 | 0.78 |
| MuVs/Missouri.USA/28.17/FH101[G] | 2017-07 | unknown | USA | Missouri | G | unknown | unknown | 0.80 |
| MuVs/Ohio.USA/2.18/FH102[G] | 2018-01 | unknown | USA | Ohio | G | unknown | unknown | 0.76 |
| MuVs/Washington.USA/4.17/FH103[G] | 2017-01-29 | Buccal Swab | USA | Washington | G | up-to-date | 34.19 | 0.56 |
| MuVs/Washington.USA/5.17/FH104[G] | 2017-02-08 | Buccal Swab | USA | Washington | G | up-to-date | 31.96 | 0.74 |
| MuVs/Washington.USA/6.17/FH105[G] | 2017-02-10 | Buccal Swab | USA | Washington | G | up-to-date | 36.53 | 0.65 |
| MuVs/Washington.USA/7.17/FH106[G] | 2017-02-14 | Buccal Swab | USA | Washington | G | not up-to-date | 27.98 | 0.67 |
| MuVs/Wisconsin.USA/7.07/FH107[G] | 2007-02 | unknown | USA | Wisconsin | G | unknown | unknown | 0.80 |
| MuVs/Wisconsin.USA/11.07/FH108[G] | 2007-03 | unknown | USA | Wisconsin | G | unknown | unknown | 0.94 |
| MuVs/Wisconsin.USA/11.07/FH109[G] | 2007-03 | unknown | USA | Wisconsin | G | unknown | unknown | 0.94 |
| MuVs/Wisconsin.USA/16.14/FH110[G] | 2014-04 | unknown | USA | Wisconsin | G | unknown | unknown | 0.92 |
| MuVs/Wisconsin.USA/20.14/FH111[G] | 2014-05 | unknown | USA | Wisconsin | G | unknown | unknown | 0.94 |
| MuVs/Wisconsin.USA/24.14/FH112[G] | 2014-06 | unknown | USA | Wisconsin | G | unknown | unknown | 0.87 |
| MuVs/Wisconsin.USA/29.14/FH113[G] | 2014-07 | unknown | USA | Wisconsin | G | unknown | unknown | 0.93 |
| MuVs/Missouri.USA/29.15/FH114[G] | 2015-07 | unknown | USA | Missouri | G | unknown | unknown | 0.84 |
| MuVs/Wisconsin.USA/42.15/FH115[G] | 2015-10 | unknown | USA | Wisconsin | G | unknown | unknown | 0.87 |
| MuVs/Wisconsin.USA/42.15/FH116[G] | 2015-10 | unknown | USA | Wisconsin | G | unknown | unknown | 0.88 |
| MuVs/Wisconsin.USA/46.15/FH117[G] | 2015-11 | unknown | USA | Wisconsin | G | unknown | unknown | 0.84 |
| MuVs/Wisconsin.USA/2.16/FH118[G] | 2016-01 | unknown | USA | Wisconsin | G | unknown | unknown | 0.91 |
| MuVs/Ohio.USA/19.16/FH119[G] | 2016-05 | unknown | USA | Ohio | G | unknown | unknown | 0.93 |
| MuVs/Wisconsin.USA/24.16/FH120[G] | 2016-06 | unknown | USA | Wisconsin | G | unknown | unknown | 0.89 |
| MuVs/Wisconsin.USA/19.16/FH121[G] | 2016-05 | unknown | USA | Wisconsin | G | unknown | unknown | 0.93 |
| MuVs/Missouri.USA/41.16/FH122[G] | 2016-10 | unknown | USA | Missouri | G | unknown | unknown | 0.85 |
| MuVs/Missouri.USA/46.16/FH123[G] | 2016-11 | unknown | USA | Missouri | G | unknown | unknown | 0.88 |
| MuVs/Missouri.USA/46.16/FH124[G] | 2016-11 | unknown | USA | Missouri | G | unknown | unknown | 0.90 |
| MuVs/Missouri.USA/50.16/FH125[G] | 2016-12 | unknown | USA | Missouri | G | unknown | unknown | 0.88 |
| MuVs/Wisconsin.USA/50.16/FH126[G] | 2016-12 | unknown | USA | Wisconsin | G | unknown | unknown | 0.88 |
| MuVs/Missouri.USA/2.17/FH127[G] | 2017-01 | unknown | USA | Missouri | G | unknown | unknown | 0.83 |
| MuVs/Ohio.USA/2.17/FH128[G] | 2017-01 | unknown | USA | Ohio | G | unknown | unknown | 0.90 |
| MuVs/Missouri.USA/7.17/FH129[G] | 2017-02 | unknown | USA | Missouri | G | unknown | unknown | 0.84 |
| MuVs/Ohio.USA/7.17/FH130[G] | 2017-02 | unknown | USA | Ohio | G | unknown | unknown | 0.89 |
| MuVs/Wisconsin.USA/15.06/FH131[G] | 2006-04 | unknown | USA | Wisconsin | G | unknown | unknown | 0.81 |
| MuVs/Ohio.USA/11.17/FH132[G] | 2017-03 | unknown | USA | Ohio | G | unknown | unknown | 0.86 |
| MuVs/Missouri.USA/11.17/FH133[G] | 2017-03 | unknown | USA | Missouri | G | unknown | unknown | 0.85 |
| MuVs/Missouri.USA/15.17/FH134[G] | 2017-04 | unknown | USA | Missouri | G | unknown | unknown | 0.85 |
| MuVs/Missouri.USA/15.17/FH135[G] | 2017-04 | unknown | USA | Missouri | G | unknown | unknown | 0.86 |
| MuVs/NorthCarolina.USA/11.17/FH136[G] | 2017-03 | unknown | USA | NorthCarolina | G | unknown | unknown | 0.84 |
| MuVs/Wisconsin.USA/15.17/FH137[G] | 2017-04 | unknown | USA | Wisconsin | G | unknown | unknown | 0.85 |
| MuVs/Missouri.USA/15.17/FH138[G] | 2017-04 | unknown | USA | Missouri | G | unknown | unknown | 0.82 |
| MuVs/Alabama.USA/15.17/FH139[G] | 2017-04 | unknown | USA | Alabama | G | unknown | unknown | 0.83 |
| MuVs/Missouri.USA/19.17/FH140[G] | 2017-05 | unknown | USA | Missouri | G | unknown | unknown | 0.84 |
| MuVs/Wisconsin.USA/19.17/FH141[G] | 2017-05 | unknown | USA | Wisconsin | G | unknown | unknown | 0.85 |
| MuVs/Washington.USA/8.17/FH142[G] | 2017-02-22 | Buccal Swab | USA | Washington | G | up-to-date | 25.33 | 0.86 |
| MuVs/Washington.USA/11.17/FH143[G] | 2017-03-15 | Buccal Swab | USA | Washington | G | up-to-date | 28.34 | 0.87 |
| MuVs/Washington.USA/12.17/FH144[G] | 2017-03-27 | Buccal Swab | USA | Washington | G | unknown | 29.66 | 0.85 |
| MuVs/Washington.USA/14.17/FH145[G] | 2017-04-06 | Buccal Swab | USA | Washington | G | up-to-date | 21.45 | 0.91 |
| MuVs/Washington.USA/15.17/FH146[G] | 2017-04-18 | Buccal Swab | USA | Washington | G | up-to-date | 27.09 | 0.84 |
| MuVs/Washington.USA/18.17/FH147[G] | 2017-05-04 | Buccal Swab | USA | Washington | G | up-to-date | 28.86 | 0.88 |
| MuVs/Washington.USA/20.17/FH148[G] | 2017-05-24 | Buccal Swab | USA | Washington | G | up-to-date | 26.59 | 0.82 |
| MuVs/Washington.USA/23.17/FH149[G] | 2017-06-11 | Buccal Swab | USA | Washington | G | unknown | 33.05 | 0.84 |
| MuVs/Washington.USA/23.17/FH150[G] | 2017-06-12 | Buccal Swab | USA | Washington | G | up-to-date | 32.33 | 0.84 |
| MuVs/Washington.USA/28.17/FH151[G] | 2017-07-12 | Buccal Swab | USA | Washington | G | up-to-date | 24.18 | 0.88 |
| MuVs/Washington.USA/16.17/FH152[G] | 2017-04-17 | Buccal Swab | USA | Washington | G | up-to-date | 27.41 | 0.85 |
| MuVs/Wisconsin.USA/41.06/FH153[G] | 2006-10 | unknown | USA | Wisconsin | G | unknown | unknown | 0.84 |
| MuVs/Wisconsin.USA/7.07/FH154[G] | 2007-02 | unknown | USA | Wisconsin | G | unknown | unknown | 0.94 |
| MuVs/Missouri.USA/33.17/FH155[G] | 2017-08 | unknown | USA | Missouri | G | unknown | unknown | 0.85 |
| MuVs/Alabama.USA/50.17/FH156[G] | 2017-12 | unknown | USA | Alabama | G | unknown | unknown | 0.85 |
| MuVs/Ohio.USA/46.17/FH157[G] | 2017-11 | unknown | USA | Ohio | G | unknown | unknown | 0.84 |
| MuVs/Washington.USA/49.16/FH158[G] | 2016-12-11 | Buccal Swab | USA | Washington | G | unknown | 30.55 | 0.84 |
| MuVs/Wisconsin.USA/28.06/FH159[G] | 2006-07 | unknown | USA | Wisconsin | G | unknown | unknown | 0.84 |
| MuVs/Washington.USA/1.17/FH160[G] | 2017-01-03 | Buccal Swab | USA | Washington | G | unknown | 24.17 | 0.87 |
| MuVs/Washington.USA/5.17/FH161[G] | 2017-01-31 | Buccal Swab | USA | Washington | G | up-to-date | 23.05 | 0.92 |
| MuVs/Washington.USA/5.17/FH162[G] | 2017-02-02 | Buccal Swab | USA | Washington | G | up-to-date | 27.08 | 0.90 |
| MuVs/Wisconsin.USA/51.15/FH163[G] | 2015-12 | unknown | USA | Wisconsin | G | unknown | unknown | 0.86 |
| MuVs/Alabama.USA/7.17/FH164[G] | 2017-02 | unknown | USA | Alabama | G | unknown | unknown | 0.80 |
| MuVs/Missouri.USA/24.17/FH165[G] | 2017-06 | unknown | USA | Missouri | G | unknown | unknown | 0.86 |
| MuVs/Washington.USA/7.17/FH166[G] | 2017-02-19 | Buccal Swab | USA | Washington | G | up-to-date | 27.96 | 0.85 |

All genomes generated for this analysis are described above. Dates are formatted as Year-month-day. Vaccination status, Ct, and sample collection type are all available for the Washington samples. Genome coverage represents the total proportion of bases in the genome with at least 20x coverage for which we were able to call a base. Sites with <20x coverage were labelled as Ns. Only samples with at least 50% non-N bases were included in the analysis.

**Supplementary File 1b: Mumps cases by age group**

| Age group | Mumps case count | 2016-2017 average population in Washington[^1^](https://paperpile.com/c/q8Le2r/SyGh) | Rate of cases per 100,000 individuals | Percentage of total cases | Counts in dataset | Percentage of dataset |
| --- | --- | --- | --- | --- | --- | --- |
| 0 - 4 | 34 | 450,847 | 7.5 | 3.8% | 8 | 7.2% |
| 5 - 9 | 104 | 462,951 | 22.5 | 11.7% | 9 | 8.2% |
| 10 - 14 | 198 | 451,485 | 43.9 | 22.3% | 28 | 25.5% |
| 15 - 19 | 214 | 455,612 | 47.0 | 24.1% | 14 | 12.7% |
| 20 - 39 | 256 | 1,980,004 | 12.9 | 28.8% | 37 | 33.6% |
| 40 - 64 | 80 | 2,348,529.5 | 3.4 | 10.0% | 13 | 11.8% |
| 65+ | 2 | 1,097,571 | 0.2 | 0.22% | 1 | 0.9% |
| Unknown | 1 | NA | NA | 0.11% | 0 | 0% |

**Supplementary File 1c: Outbreak characteristic and dataset composition**

|  | counts in outbreak (%) | Count in dataset (%) |
| --- | --- | --- |
| Up-to-date vaccination | 574 (65%) | 64 (58%) |
| Not up-to-date vaccination | 86 (9.7%) | 13 (12%) |
| Unknown vaccination status | 229 (26%) | 33 (30%) |
| Marshallese | 465 (52%) | 57 (52%) |
| Not Marshallese | 424 (48%) | 53 (48%) |
| **Total** | **889** | **110** |

**Supplementary File 1d: Raw tip counts used for divergence tree transmission metric**

| Metadata category | Total tips | Tips labeled as basal | Tips labeled as terminal |
| --- | --- | --- | --- |
| Vaccination not up-to-date | 13 | 4 | 9 |
| Vaccination up-to-date | 63 | 30 | 34 |
| Vaccination status unknown | 32 | 12 | 20 |
| Age < 20 years | 59 | 29 | 30 |
| Age ≥ 20 years | 50 | 17 | 33 |
| Marshallese | 57 | 32 | 25 |
| Not Marshallese | 52 | 14 | 38 |

**Supplementary References**

1. [Estimates of April 1 population by age, sex, race and Hispanic origin.](http://paperpile.com/b/q8Le2r/SyGh) <https://www.ofm.wa.gov/washington-data-research/population-demographics/population-estimates/estimates-april-1-population-age-sex-race-and-hispanic-origin>[.](http://paperpile.com/b/q8Le2r/SyGh)
