# Supplementary material for: Repeated introductions and intensive community transmission fueled a mumps virus outbreak in Washington State
Source: eLife. 2021 Apr 19;10:e66448. doi: 10.7554/eLife.66448 (PMC8079146; doi:10.7554/eLife.66448)
Supplement: Transparent reporting form [file elife-66448-transrepform.docx]

***eLife’s* transparent reporting form**

We encourage authors to provide detailed information *within their submission* to facilitate the interpretation and replication of experiments. Authors can upload supporting documentation to indicate the use of appropriate reporting guidelines for health-related research (see [EQUATOR Network](about:blank)), life science research (see the BioSharing Information Resource), or the [ARRIVE guidelines](about:blank) for reporting work involving animal research. Where applicable, authors should refer to any relevant reporting standards documents in this form.

If you have any questions, please consult our Journal Policies and/or contact us: [editorial@elifesciences.org](about:blank).

**Sample-size estimation**

- You should state whether an appropriate sample size was computed when the study was being designed
- You should state the statistical method of sample size computation and any required assumptions
- If no explicit power analysis was used, you should describe how you decided what sample (replicate) size (number) to use

Please outline where this information can be found within the submission (e.g., sections or figure legends), or explain why this information doesn’t apply to your submission:

A detailed description of how samples were selected for sequencing and inclusion in the study, in addition to ensuring that our dataset closely mirrored the distribution of epidemiologic groups affected by the outbreak is in the Methods under “Sample collection and IRB approval.” A comparison between the distribution of samples in our dataset to the overall outbreak is shown in Supplemental Table 3.

**Replicates**

- You should report how often each experiment was performed
- You should include a definition of biological versus technical replication
- The data obtained should be provided and sufficient information should be provided to indicate the number of independent biological and/or technical replicates
- If you encountered any outliers, you should describe how these were handled
- Criteria for exclusion/inclusion of data should be clearly stated
- High-throughput sequence data should be uploaded before submission, with a private link for reviewers provided (these are available from both GEO and ArrayExpress)

Please outline where this information can be found within the submission (e.g., sections or figure legends), or explain why this information doesn’t apply to your submission:

For all Bayesian phylogenetic reconstructions of population structure/dynamics, we ran 3 independent MCMC such that each analysis was run in triplicate. Details are included in the Methods under “Inference of community transmission dynamics using a structured coalescent model.” For the rarefaction analysis, we performed each subsampling 10 times, as detailed in the Methods under “Rarefaction analysis to estimate transmission clusters.”

**Statistical reporting**

- Statistical analysis methods should be described and justified
- Raw data should be presented in figures whenever informative to do so (typically when N per group is less than 10)
- For each experiment, you should identify the statistical tests used, exact values of N, definitions of center, methods of multiple test correction, and dispersion and precision measures (e.g., mean, median, SD, SEM, confidence intervals; and, for the major substantive results, a measure of effect size (e.g., Pearson's r, Cohen's d)
- Report exact p-values wherever possible alongside the summary statistics and 95% confidence intervals. These should be reported for all key questions and not only when the p-value is less than 0.05.

Please outline where this information can be found within the submission (e.g., sections or figure legends), or explain why this information doesn’t apply to your submission:

For each summary tree we show in the manuscript, each internal node is labelled with the posterior probability that the internal node state is labelled within the group of interest, as well as a mean trait (date, geographic location, or community membership) and 95% highest posterior density of the estimated trait. In each displayed Bayesian phylogeny, this uncertainty is shown with internal node opacity corresponding to posterior probability. Wherever possible, we have also elected to plot 95% highest posterior density distributions (Fig. 3b, 5c, and Supplemental Figures 4c, 7b) and individual values (Fig. 4b, 5b, and Supplemental Figures 3, 4b, 5b, and 7a), rather than summary statistics in order to display the spread and uncertainty in the data points. Details regarding how the summary tree and 95% highest posterior densities were calculated are included in the Methods under “Phylogenetic analysis of full North American mumps genomes” and “Inference of community transmission dynamics using a structured coalescent model”. For the logistic regression presented in Table 1, the full model details and p-value determinations are outlined in the Methods under “Testing for descendants in divergence trees.”

(For large datasets, or papers with a very large number of statistical tests, you may upload a single table file with tests, Ns, etc., with reference to sections in the manuscript.)

**Group allocation**

- Indicate how samples were allocated into experimental groups (in the case of clinical studies, please specify allocation to treatment method); if randomization was used, please also state if restricted randomization was applied
- Indicate if masking was used during group allocation, data collection and/or data analysis

Please outline where this information can be found within the submission (e.g., sections or figure legends), or explain why this information doesn’t apply to your submission:

A detailed description of how samples were selected for sequencing and inclusion in the study, in addition to ensuring that our dataset closely mirrored the distribution of epidemiologic groups affected by the outbreak is in the Methods under “Sample collection and IRB approval.” A comparison between the distribution of samples in our dataset to the overall outbreak is shown in Supplemental Table 3.

**Additional data files (“source data”)**

- We encourage you to upload relevant additional data files, such as numerical data that are represented as a graph in a figure, or as a summary table
- Where provided, these should be in the most useful format, and they can be uploaded as “Source data” files linked to a main figure or table
- Include model definition files including the full list of parameters used
- Include code used for data analysis (e.g., R, MatLab)
- Avoid stating that data files are “available upon request”

Please indicate the figures or tables for which source data files have been provided:

We have made all code and raw data available as described in the Methods under the section, “Data and Code Availability.” We also provide a complete list of sequenced genomes with limited metadata in Supplemental Table 1. We are unable to publish complete metadata, as it is considered identifiable.
